# Exposure to Deoxynivalenol During Pregnancy and Lactation Enhances Food Allergy and Reduces Vaccine Responsiveness in the Offspring in a Mouse Model

**DOI:** 10.3389/fimmu.2021.797152

**Published:** 2021-12-17

**Authors:** Negisa Seyed Toutounchi, Saskia Braber, Belinda van’t Land, Suzan Thijssen, Johan Garssen, Aletta D. Kraneveld, Gert Folkerts, Astrid Hogenkamp

**Affiliations:** ^1^ Division of Pharmacology, Utrecht Institute for Pharmaceutical Sciences, Faculty of Science, Utrecht University, Utrecht, Netherlands; ^2^ Danone Nutricia Research, Utrecht, Netherlands; ^3^ Center of Translational Immunology, Wilhelmina Children’s Hospital, University Medical Center Utrecht, Utrecht, Netherlands

**Keywords:** Deoxynivalenol, developmental immunotoxicity, food allergy, vaccination, pregnancy, lactation

## Abstract

Deoxynivalenol (DON), a highly prevalent contaminant of grain-based products, is known to induce reproductive- and immunotoxicities. Considering the importance of immune development in early life, the present study investigated the effects of perinatal DON exposure on allergy development and vaccine responsiveness in the offspring. Pregnant mice received control or DON-contaminated diets (12.5 mg/kg diet) during pregnancy and lactation. After weaning, female offspring were sensitized to ovalbumin (OVA) by oral administration of OVA with cholera toxin (CT). Male offspring were injected with Influvac vaccine. OVA-specific acute allergic skin response (ASR) in females and vaccine-specific delayed-type hypersensitivity (DTH) in males were measured upon intradermal antigen challenge. Immune cell populations in spleen and antigen-specific plasma immunoglobulins were analyzed. In female CT+OVA-sensitized offspring of DON-exposed mothers ASR and OVA-specific plasma immunoglobulins were significantly higher, compared to the female offspring of control mothers. In vaccinated male offspring of DON-exposed mothers DTH and vaccine-specific antibody levels were significantly lower, compared to the male offspring of control mothers. In both models a significant reduction in regulatory T cells, Tbet^+^ Th1 cells and Th1-related cytokine production of the offspring of DON-exposed mothers was observed. In conclusion, early life dietary exposure to DON can adversely influence immune development in the offspring. Consequently, the immune system of the offspring may be skewed towards an imbalanced state, resulting in an increased allergic immune response to food allergens and a decreased immune response to vaccination against influenza virus in these models.

## 1 Introduction

Pregnancy and lactation represent crucial periods in the development of the newborn’s immune system (1). A wide range of contaminants present in maternal food and environment during these periods can interfere with the process of immune programming, leading to long-term or permanent changes in the offspring (1, 2). The most critical immune maturational events occur during the early life stages (3, 4). Therefore, any immune disturbance in this early period can result in altered immune function, and can even have significant long-term consequences for the offspring (5, 6). A strong connection between developmental immunotoxicity and the elevated risk for immune related disorders has been suggested. Diseases such as childhood asthma and allergies (7), chronic otitis media, type-1 diabetes, childhood leukemia and pediatric celiac disease are all related to disturbed and imbalanced immune capacity during early stages of immune development (8). During pregnancy, the maternal immune system shifts towards a distinct and more tolerogenic state by down regulating Th1-mediated immune responses and increasing production of Th2-mediated cytokines, in order to prevent Th1-driven rejection of the semi-allogeneic fetus (9). Similarly, the immune system of the newborn after birth is unbalanced and skewed toward Th2 responses (7). Moreover, the infant is born with an immature (though functional) immune system which will naturally mature after exposure to different antigens and infections during the first months and years of life. Any prenatal or neonatal environmental factors that interfere with the immune programming in early life, can impose a risk for allergy development and diminished host resistance to disease.

Mycotoxins are among the most important and highly prevalent nutritional contaminants with well-established immunotoxic properties (10). They are naturally produced as secondary metabolites of different fungal species, which can contaminate a wide range of agricultural products, especially cereal and grain-based food (11). As a result of the high prevalence of fungal contamination in the food chain, mycotoxin exposure is almost inevitable. Epidemiological studies from different geographical regions have shown that pregnant women and newborns are highly exposed to different mycotoxins (2). Deoxynivalenol (DON), a trichothecene mycotoxin produced by different *Fusarium* fungi species, is one of the most prevalent mycotoxins occurring in human food (12). DON exhibits intestinal, neurological, reproductive and immunotoxicity (13). Considerable concentrations of DON were detected in urine samples of pregnant women in different geographical regions, some exceeding the proposed maximum tolerable daily intake (TDI; 1 µg/kg of body weight per day) (14, 15). DON can pass through the placenta and reach the fetus during pregnancy (16–18), and is transferred into the milk during lactation (18, 19), which signifies the importance of exploring the potential adverse effects of DON exposure in newborns. Direct exposure to DON induces immunotoxicity, even with very low doses (20). Depending on the concentration and duration of exposure, DON can induce both immunosuppressive and immunostimulatory effects (21). Immunotoxicity of DON might be induced through oxidative stress and DNA damage (22), and inhibition of lymphocyte proliferation (23). Previously, it was shown that sensitizing mice by intragastric gavage of whey proteins in combination with DON (100 µg per mouse), enhances allergic reactions to whey proteins, possibly by disturbing the integrity of the intestinal epithelial barrier as an adjuvant, and inducing cell stress, resulting in the initiation of Th2 responses and allergic reactions (24). Moreover, a compromised resistance to enteric and pulmonary reovirus infections was reported after DON exposure in mice models (25, 26), possibly due to suppression of type-1 IFN-mediated responses.

Although immunomodulatory effects of direct exposure to DON are extensively studied (10, 20–22), information about the immunotoxicity of exposure to DON during pregnancy is scarce (27). Intravenous administration of DON in pregnant pigs at the end of gestation was shown to alter mRNA expression of IFN-γ, IL-17, IL-2, and TNF-α, in the blood leukocytes of the piglets, and induce significant decrease in the population of regulatory T cells, and an increase in cytotoxic and γδ T cells in blood samples of these piglets, 1-3 weeks after birth (27). These changes indicate a disturbed and unbalanced immune system in piglets of DON-exposed mothers after birth. To our knowledge, there are no data available on the outcome of early life DON exposure during pregnancy and lactation on the development of immune-related disease or vaccination responses in the later stages of life. Considering the potential immunotoxicity of DON and its effect on Th1/Th2 immune responses, in the current study the effect of maternal exposure to DON during pregnancy and lactation on the immune maturation and Th1/Th2 balance in the offspring was investigated using the ovalbumin (OVA)-induced food allergy model to examine the allergic response, and the Influenza-vaccination model to assess the vaccination response.

## 2 Materials and Methods

### 2.1 Animals and Diets

Eight-week-old female and male C3H/HeOuJ mice were purchased from Charles River Laboratories and housed in the animal facility of Utrecht University at controlled temperature (21 ± 2°C) and humidity (50–55%), with a reversed 12:12 hours light/dark cycle (lights on from 7.00 pm till 7.00 am) and with *ad libitum* access to food and tap water. All experimental procedures were carried out during the dark cycle and all repeated measurements were performed at the same time block of the day. Animals were kept in makrolon cages (22 cm×16 cm×14 cm, floor area 350 cm2, Tecnilab- BMI, Someren, the Netherlands) with wood-chip bedding (Tecnilab- BMI, Someren in the Netherlands), and tissues (VWR, the Netherlands) and shelters were available as cage enrichments. The animals received standard diets (pelleted food, AIN-93G, Ssniff Spezialdiäten, Soest, Germany) and routine care for a week upon arrival in the animal facility before the start of breeding. This study was conducted in accordance with institutional guidelines for the care and use of laboratory animals established by the Animal Ethics Committee of the Utrecht University, and all animal procedures related to the purpose of the research were approved under license of the national competent authority, securing full compliance the European Directive 2010/63/EU for the use of animals for scientific purposes.

Semi‐purified AIN‐93G soy protein‐based diet was composed [the details of the diet content have been previously described (28)] and mixed by DON (FERMENTEK Ltd, Jerusalem, Israel) at concentration of 12.5 mg per kg of diet, by Ssniff Spezialdiäten GmbH (Soest, Germany).

### 2.2 Study Design

**Figure 1:**
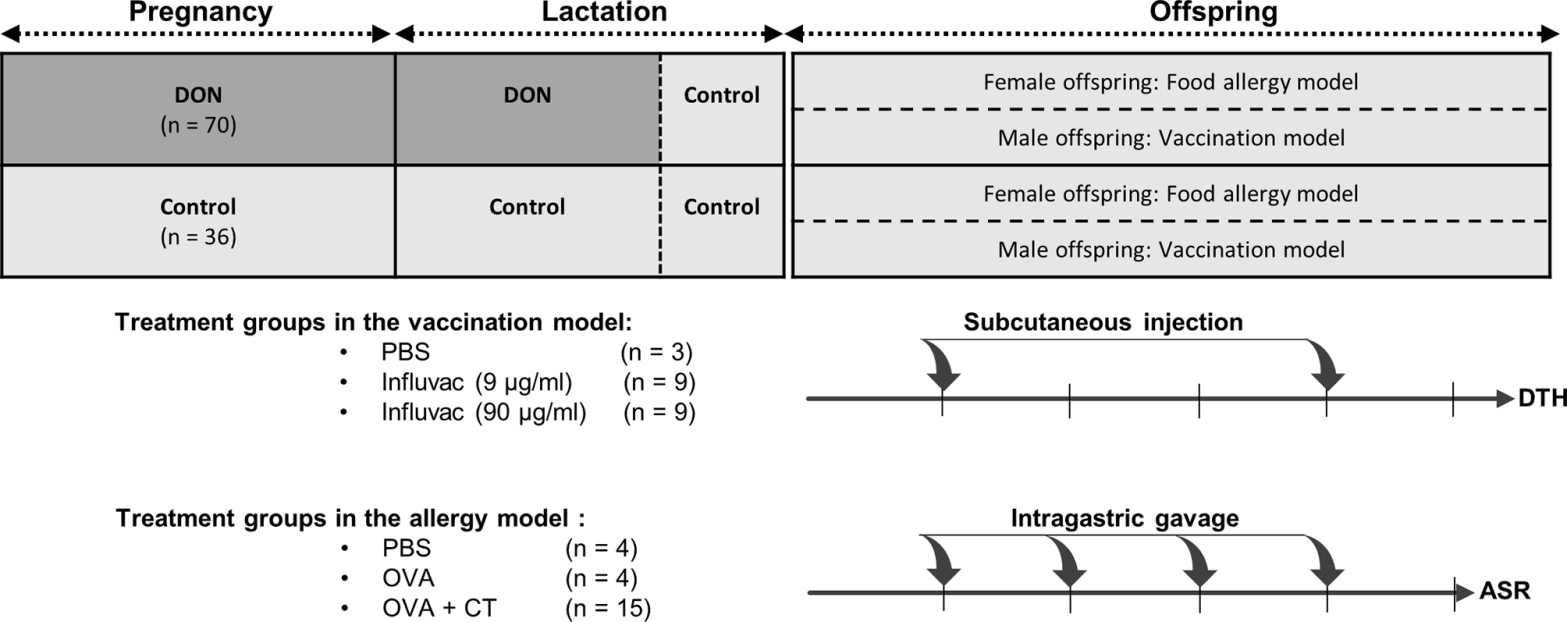
Schematic overview of study design. Female mice received either control (AIN93) or Deoxynivalenol-contaminated (DON) diets during pregnancy and lactation period, until a week before weaning. The offspring received control diet right after weaning until the end of the experiment, and were used in either 1) vaccination model (male offspring), receiving 2 injections of phosphate-buffered saline (PBS) or Influvac; or 2) food allergy model (female offspring), receiving intragastric gavage of PBS, ovalbumin (OVA) or OVA with cholera toxin (CT). The number of animals in each group is indicated as n. At the end of the experiment, the animals in vaccination model underwent delayed-type hypersensitivity (DTH) assay, and the animals in food allergy model underwent acute allergic skin response (ASR) assay.

A schematic overview of the study design is shown in **Figure 1**. After an acclimatization period, animals were weighed and mated by putting one male mouse together with 2 female mice in the home-cage of the females, for 4 days. After mating, the male mouse was removed from the cage and female mice were randomly assigned to a dietary group, in a way that the average weight of the animals in each dietary group was not significantly different. Control group received standard AIN-93G diet, and DON group received AIN-93G diet containing 12.5 mg DON (per kg of diet) throughout the entire period of pregnancy and the first 2 weeks of the lactation period. Results of a preliminary experiment in our group showed that concentration of 12.5 mg/kg of DON is not inducing acute toxicity, or significant weight loss in mice (unpublished data). Although the concentration of DON used in this study is above the maximum permitted levels of DON in different cereals and cereal-based products (1-2 mg/kg of food) (29), numerous studies have reported a considerable percentage of food products exceeding this safety level (30–32); DON concentration in several commodities were reported to be as high as 20 mg/kg of food (33). Moreover, consuming high amounts of cereals and cereal-based products, and combined consumption of a wide variety of DON-contaminated food can lead to a significantly higher exposure levels in specific populations (33). Thus, the concentration of DON used in this study can be considered relevant to the human exposure in the areas with high DON contamination.

In order to prevent any direct digestion of DON by the offspring, all lactating mothers received control diet during the last week of lactation. Breeding female mice were weighed on day 0, before starting DON-diet, and at end of lactation period, to evaluate the weight gain in these animals. After weaning, all offspring were put on control diet. After separating the males from the females, the offspring from either control dams or DON-treated dams were briefly grouped together, after which the offspring were randomly allocated to the different treatment groups. Both female and male offspring were housed as 4 and 3 animals per cage, respectively. Female offspring were used in ovalbumin (OVA) food allergy model, and male offspring were used in vaccination model. Details of the sample size calculation and total number of animals in each group is explain in the statistical analysis.

#### 2.2.1 Food Allergy Model

One week after weaning, 4-week-old female offspring were sensitized to OVA according to the previously established OVA-specific food allergy model (34). Briefly, the animals received oral gavage of either phosphate-buffered saline (PBS; control group), OVA (OVA-sensitized group; 20 mg in 500 μL PBS; grade V; Sigma-Aldrich, Zwijndrecht, The Netherlands) or OVA together with cholera toxin (CT, 10 μg in 500 μL PBS) as an adjuvant (OVA+CT-sensitized allergy group), once a week for 4 weeks.

One week after the last sensitization, acute allergic skin responses (ASR) were measured by intradermal challenge, as described below. Six hours after the intradermal challenge, 9-week-old mice were challenged orally with OVA (100 mg in 500 μL) and 15 hours after the oral challenge, blood samples were collected by orbital extraction under isoflurane-induced inhalation anesthesia, followed by cervical dislocation. Thereafter, tissue samples were collected for *ex vivo* analyses.

##### 2.2.1.1 Acute Allergic Skin Response

Acute allergic skin response (ASR) was measured by intradermal injection of OVA (1 pg in 25 μL PBS) into the pinna of left ear and 25 μL PBS into the pinna of right ear, under isoflurane-induced inhalation anesthesia. Changes in ear thickness, as a readout for Th2-mediated allergic reaction, was measured 1 hour after injection by using a digital micrometer (Mitutoyo Digimatic 293561, Veenendaal, The Netherlands). Based on earlier studies, ear swelling reaches to an optimum level 1 hour after the antigen challenge (35). The ASR was calculated by following formula:


ASR=[right ear (thickness at 1 h−thickness at 0 h)]−(left ear (thickness at 1 h−thickness at 0 h)

##### 2.2.1.2 Anaphylactic Shock Score and Body Temperature

To evaluate the allergic response in the ovalbumin-sensitized offspring, anaphylactic reactions, such as scratching around nose or swollen eyes (**Table 1**) and drop in body temperature as clinical shock symptoms were determined 45 min after intradermal challenge. To establish the severity of the shock, a previously established anaphylactic scoring table was used (34). Animals were closely monitored after intradermal challenge and body temperature was detected using a rectal thermometer (Terumo, Leuven, Belgium). In case of severe shock symptoms and drop in body temperature, animals were placed on heating pads.

**Table 1:** The list of anaphylactic symptoms used as the scoring criteria in food allergy model.

Score	Anaphylactic shock symptoms
0	no symptoms
1	scratching around nose and/or mouth
2	swollen eyes and/or mouth, piloerection, reduced mobility, increased breath frequency
3	shortness of breath and/or increased breath frequency, bluish color around mouth and tail, further reduced/painful mobility
4	no mobility following stimulation, convulsions

##### 2.2.1.3 Serum Concentration of Specific Antibodies and Mouse Mast Cell Protease‐1 (mMCP-1)

Blood samples collected from female offspring were centrifuged (12000 RCF for 10 min) to collect the serum and were stored at −20°C until analysis. OVA-specific IgG1, IgG2a and IgE were measured in serum samples of female offspring using enzyme-linked immunosorbent assay (ELISA), as described previously (36). Concentrations in test sera were calculated in arbitrary units (AU), relative to the standard curve of pooled plasma. Additionally, concentration of murine Mast cell protease-1 (mMCP‐1) was measured in sera collected from female offspring, using a mMCP‐1 Ready‐SET‐Go!^®^ ELISA (eBioscience, San Diego, CA, USA) according to the manufacturer’s instructions.

##### 2.2.1.4 Splenocyte Re-Stimulation Assay

Re-stimulation of splenocytes for the analysis of *ex vivo* cytokine production was carried out as described earlier (36). Splenocytes were isolated by smashing the spleen samples through 70‐µm nylon cell strainer, and were counted and re-suspended in RMPI-1640 medium supplemented with 10% FCS, 100 U/mL penicillin and 100 mg/mL streptomycin (culture medium) with or without 50 µg/mL OVA. After 5 days of culturing cells in 96-well U-bottom culture plates at 37°C in a humidified environment containing 5% CO2, supernatants were harvested and analyzed for determining the concentration of interleukin (IL)-4, IL-6, IL-10, IL-12p70, IL-13, IL-27, tumor necrosis factor (TNF)-α and interferon (IFN)-γ using ProcartaPlex multiplex protein assay kit (Invitrogen, Thermo Fisher Scientific, Waltham, MA, USA) according to manufacturer’s instructions.

##### 2.2.1.5 qPCR Intestine

For mRNA isolation, proximal small intestine samples collected from mice were immediately frozen on dry ice and kept at −80°C until analysis. Tissue samples were weighed and homogenized in lysis buffer with 1:1 (w/v) ratio. Total RNA was isolated after DNAse treatment, using SV Total RNA Isolation System (Promega Corporation, Madison, WI, USA), based on the manufacturer’s instructions. Subsequently, the iScript cDNA Synthesis kit (Bio-Rad Laboratories, Hercules, CA, USA) was used to reverse-transcribe the RNA into cDNA, using the T100 thermal cycler (Bio-Rad Laboratories, Hercules, CA, USA).

Selected primers for zonula ocludence-1 (ZO-1), claudin-4 (CLDN-4), occludin (OCLD) and E-cadherin (Bio-Rad Laboratories, Hercules, CA, USA), together with iQSYBR Green Supermix (Bio-Rad Laboratories, Hercules, CA, USA) were used for qPCR (the primer efficiency for each tested primer was between 96 - 105%), and amplification was performed according to the manufacturer’s instructions using the CFX96 Touch™ Real-Time PCR Detection System (Bio-Rad Laboratories, Hercules, CA, USA). The mRNA for each gene was normalized using the geometric mean of 2 reference genes, β-actin and glyceraldehyde 3-phosphate dehydrogenase (GAPDH), which are previously shown to be stable after DON exposure (37), and relative mRNA expression for each mouse was depicted as a fold change of the average of control group.

#### 2.2.2 Vaccination Model

The 4-week-old male offspring received vaccination one week after weaning, using Influvac (Abbott Biologicals B.V., Weesp, The Netherlands) from season 2015/2016, as previously described (38). The mice received the primary and booster vaccinations by subcutaneous injections of 100 µL undiluted Influvac (containing hemagglutinin (HA) and neuraminidase antigens of three strains of influenza virus, in a dose equivalent to 30 µg/mL HA per strain, in total 90 µg/mL HA), or 10-times diluted vaccine (9 µg/mL HA). The booster vaccination was given 21 days after the primary vaccination. Sham group (negative control) which received injections of 100 µL PBS instead of vaccine was used to demonstrate the specificity of vaccine-induced response. Different doses of Influvac were tested in order to determine which dose would generate the immune responses with a larger effect size, so that the potential modulations by DON could be detected. Delayed-type hypersensitivity (DTH) was measured 9 days after booster vaccination, as described below. A day after DTH measurement, blood samples were collected by orbital extraction under inhalation anesthesia, followed by cervical dislocation. Tissue samples were collected for *ex vivo* analyses.

##### 2.2.2.1 Antigen Specific Delayed-Type Hypersensitivity Reactions

DTH reaction, as a model for cellular Th1-mediated immune reactivity, was determined 9 days after the booster vaccination, as described previously (38). Undiluted Influvac (20 µL) and PBS were injected intradermally into the ear pinnae of right and left ears, respectively, under isoflurane induced anesthesia. Ear thickness was measured in duplicate using digital-micrometer before injection and 24 hours thereafter, and the change in ear thickness was calculated by following formula:


DTH=[right ear (thickness at 24 h−thickness at 0 h)]−(left ear (thickness at 24 h−thickness at 0 h)

##### 2.2.2.2 Re-Stimulation of Splenocytes With Vaccine-Loaded Bone Marrow-Derived Dendritic Cells (BMDCs)

Bone marrow cells were isolated from femurs and tibias of healthy and untreated 5-week-old male mice born to control-fed mothers. Collected cells were cultured in RPMI 1640 medium (Gibco) supplemented with 10% FBS, 100 U/mL penicillin/streptomycin, 10 mM HEPES, 1 mM sodium pyruvate, and Eagles minimum essential medium (MEM) non-essential amino acids (all from Gibco Life Technologies) in the presence of 10 ng/mL GM-CSF (Prosepec, The Netherlands) for 6 days to obtain immature BMDC (iDC) (38). Induced iDCs were loaded with Influvac vaccine at a concentration of 0.9 µg/mL and incubated for 24 hours at 37°C, 5% CO2 to obtain matured DCs. DCs treated with medium were used as negative control. Spleen samples collected from vaccinated mice were smashed and splenocytes were isolated and resuspended in above mentioned supplemented culture medium, without GM-CSF. Freshly prepared splenocytes were cocultured with matured DCs at 10:1 ratio, in 96-well U-bottom culture plates for 5 days at 37°C, 5% CO2. Cell supernatants were collected and analyzed for the concentration of cytokines using ProcartaPlex multiplex protein assay kit (Invitrogen) according to manufacturer’s instructions.

##### 2.2.2.3 Serum Concentration of Specific Antibodies

Blood samples collected from both male offspring were centrifuged (12000 RCF for 10 min) to collect the serum and were stored at −20°C until analysis. The serum samples were used to determine concentration of vaccine-specific immunoglobulin (Ig)G1 and IgG2a, according to the methods described previously (38, 39) by enzyme-linked immunosorbent assay (ELISA). Concentrations in test sera were calculated in arbitrary units (AU), relative to the standard curve of pooled plasma.

### 2.3 Flow Cytometric Analysis of Spleen

Spleen samples collected from both male and female offspring were used for analyzing different immune cell populations by means of flow cytometry. Fresh splenocytes were isolated from spleens by methods described previously (36). A complete list of labeled monoclonal antibodies used for staining the cells are shown in **Supplementary Table 1**. Cell viability was assessed by means of a fixable viability dye eFluor^®^ 780 (eBioscience). For detecting intracellular transcription factors, Foxp3 Staining Buffer Set (eBioscience) were used for fixing and permeabilizing the cells, according to manufacturer’s protocol. Results were collected with BD FACSCanto II flow cytometer (Becton Dickinson, Franklin Lakes, NJ, USA) and analyzed with FlowLogic software (Inivai Technologies, Mentone, VIC, Australia).

### 2.4 Statistical Analysis

All data were analyzed by GraphPad Prism 8.0 software (GraphPad Software, San Diego, CA, USA). For normally distributed data one-way ANOVA test was used, followed by a Bonferroni’s multiple comparison *post hoc* test for selected comparisons, and for not normally distributed and non‐parametric data Kruskal‐Wallis test was performed followed by Dunn’s multiple comparisons test. Unpaired t test was used for comparing 2 means and in order to compare the ratio Fisher’s exact test for differences between proportions was performed. Data are presented as mean ± SEM. *p < 0.05, **p < 0.01 and ***p < 0.001 were considered statistically significant.

The required sample size was calculated separately for allergy and vaccination models, using G*Power v3.1.9. In allergy model, sample size was calculated based on ASR data available from previous experiments (24). Control and OVA-treated groups were used to confirm the validity of the model, therefore based on data available from previous studies, a group size of n=4 was proven to be sufficient. The required number of animals in groups treated with OVA+CT was calculated as n=15, based on considering an outcome of 30% change in ASR as minimum relevant difference. The required sample size for vaccination model was calculated based on DTH data available from previous vaccination experiments (38). Sham-treated groups were used for model validation, therefore a group size of n=3 was considered sufficient. The number of animals in vaccinated groups was calculated as n=9, based on considering an outcome of 10% change in ear thickness as minimum relevant difference. The power was set on 0.9 and α was corrected to the number of relevant comparisons. Based on the number of offspring required for both allergy and vaccination models, number of breeding female mice was calculated. Breeding success for female mice fed with control and DON-contaminated diets was calculated as 40% and 30%, respectively, based on results of a preliminary experiment in our group (unpublished data).

## 3 Results

Exposure to DON during pregnancy adversely affected the pregnancy outcome in mice. The breeding success in the control group receiving AIN93G diet was 39% (14 out of 36 mice) and in the female mice receiving DON-contaminated diet throughout the pregnancy period was 31% (22 out of 70 mice), though the difference was not statistically significant. The average litter size in DON-exposed mice was significantly smaller than control group (2.2 and 4.8, respectively, p < 0.05), but the ratio of male to female offspring was not significantly different between DON-exposed and control groups (0.7 and 1.1, respectively). The average weight gain from day 0 of pregnancy until the end of lactation period was 4.4 g in control-fed mice (n=14, SD=1.1) and 3.9 g in DON-fed mice (n=22, SD= 0.9), though the difference between groups was not statistically significant.

### 3.1 Maternal Exposure to DON Leads to Enhanced Allergic Immune Responses to OVA in the Offspring

**Figure 2:**
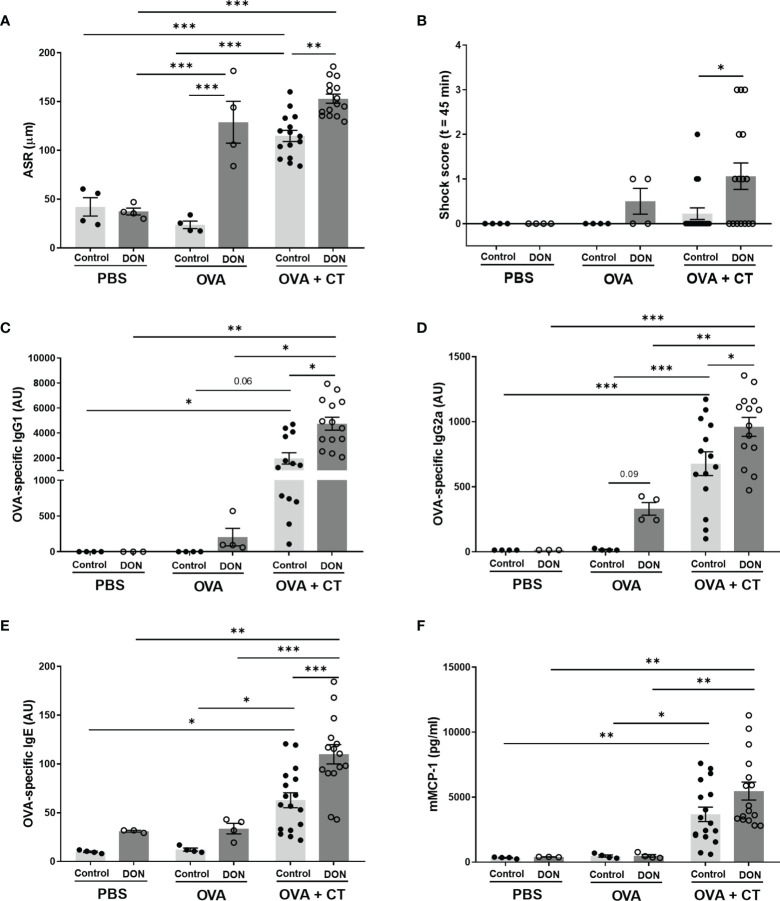
Maternal deoxynivalenol (DON) exposure enhanced ovalbumin (OVA)-specific allergic response and serum immunoglobulins (Ig) in the female offspring. Pregnant mice fed either a control or DON-contaminated diet (12.5 mg/kg) during pregnancy and lactation period. Female offspring received oral sensitizations with either phosphate-buffered saline (PBS), OVA, or OVA with cholera toxin (CT) after weaning. A week after the last sensitization, **(A)** OVA-specific acute allergic skin response (ASR), measured as changes in ear thickness 1 hour after intradermal challenge; and **(B)** accompanying anaphylactic shock score was determined 45 minutes after intradermal challenge with OVA. Serum levels of **(C)** OVA-specific IgG1, **(D)** IgG2a, **(E)** IgE, and **(F)** murine Mast cell protease-1 (mMCP-1) were measured 15 hours after oral gavage with ovalbumin. Data are presented as mean ± SEM. *p < 0.05, **p < 0.01 and ***p < 0.001 indicate statistical differences.

Results of the acute allergic skin response (ASR), anaphylactic shock scores and serum concentration of OVA-specific immunoglobulins are shown in **Figure 2**. In all animals sensitized with OVA+CT, ASR was significantly higher compared to PBS-treated mice (**Figure 2A**), and OVA-specific IgG1, IgG2a, IgE and mMCP-1 levels in the serum was significantly increased (**Figures 2C–F**), compared to non-(PBS) or OVA-sensitized mice. Maternal exposure to DON significantly enhanced ASR (**Figure 2A**, p<0.01), and increased OVA-specific IgG1 and IgG2a (**Figures 2C, D**, p<0.05) and IgE (**Figure 2E**, p<0.001) levels in OVA+CT-sensitized animals, while no significant effect of DON on mMCP-1 concentrations was observed (**Figure 2F**). Scoring the animals for development of shock symptoms 45 minutes after intradermal challenge revealed that 20% of OVA+CT-sensitized mice born to control-fed mothers showed mild-to-moderate shock signs, while in OVA+CT-sensitized mice born to DON-exposed mothers 60% of them had anaphylactic shock, with 20% showing severe symptoms (**Supplementary Table 2**). Thus, more animals developed anaphylactic shock symptoms, and severity of symptoms was enhanced in animals born to DON-exposed mothers, compared to those from control mothers (**Figure 2B**, p<0.05). No significant difference in body temperature was observed between treatment and dietary groups (**Supplementary Figure 1**).

Interestingly, OVA-sensitized mice (without CT) born to DON-exposed mothers also developed shock symptoms and showed high ASR and vaccine-specific IgG2a production compared to non-sensitized groups and OVA-sensitized group born to control mothers (**Figures 2A, D**), thought the differences in IgG2a levels are not significant due to small sample size (p=0.09). This indicates higher susceptibility of these animals to develop allergic response without receiving CT as adjuvant.

### 3.2 Maternal DON Exposure Induces Limited Effects on mRNA Expression of Junctional Proteins in the Duodenum of the Offspring

Direct exposure to DON is known to disrupt intestinal barrier integrity through changing the expression and localization of tight junction proteins (37). Therefore, to gain more insight into possible intestinal integrity changes that might contribute to OVA allergic response, gene expression of junctional proteins in duodenum samples were measured in mice after oral OVA challenge. The expression of the target genses were normalized using 2 references genes, β-actin and GAPDH. There was no significant difference between different groups on mRNA expression of ZO-1, occludin (OCLD), E-cadherin and of claudin-4 (**Supplementary Figures 2A–D**).

### 3.3 Influvac-Specific Immune Responses Are Attenuated in Offspring Born to DON-Exposed Mothers

**Figure 3:**
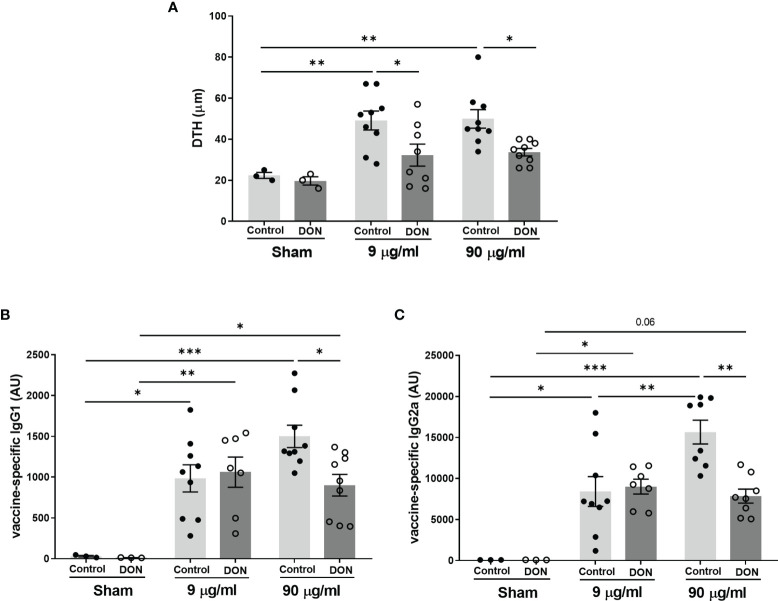
Maternal deoxynivalenol (DON) exposure reduced vaccine-specific immune responses in the male offspring. Pregnant mice fed either a control or DON-contaminated diet (12.5 mg/kg) during pregnancy and lactation period. Male offspring received subcutaneous injections of either phosphate-buffered saline (PBS, sham) or Influvac (9 and 90 µg/mL) after weaning. 10 days after the second injection, **(A)** vaccine antigen-specific delayed-type hypersensitivity (DTH) was measured as changes in ear thickness 24 hours after intradermal challenge in Influvac; and **(B)** vaccine antigen-specific immunoglobulin (Ig)G1 and **(C)** IgG2a levels were measured in serum. Data are presented as mean ± SEM. *p < 0.05, **p < 0.01 and ***p < 0.001 indicate statistical differences.

Results of Influvac-induced DTH reaction, as a model for *in vivo* cellular Th1 dependent immunity, showed a significant antigen-specific response to Influvac in vaccinated mice born to control mothers (**Figure 3A**, p<0.01) and there was no significant difference between the 2 doses of influenza vaccine(9 and 90 µg/mL). However, the DTH response was significantly diminished in vaccinated mice born to DON-exposed mothers, compared to vaccinated mice from control mothers at both tested doses of vaccine (p<0.05).

As expected, all vaccinated mice had a significantly higher production of Influvac-specific IgG1 and IgG2a, compared to sham-treated groups (**Figures 3B, C**). In vaccinated mice born to control mothers, administration of undiluted vaccine induced higher Influvac-specific IgG production compared to the diluted vaccine, while this effect was not observed in offspring of DON-exposed mice. When comparing groups receiving the higher dose of vaccine, mice born to DON-fed group had significantly lower serum levels of Influvac-specific IgG1 and IgG2a compared to those from the control-fed group (p<0.05 and p<0.01, respectively).

### 3.4 Maternal DON Exposure Reduces Splenic Tbet^+^ T Helper 1 Cells and Regulatory T Cells in the Offspring

**Figure 4:**
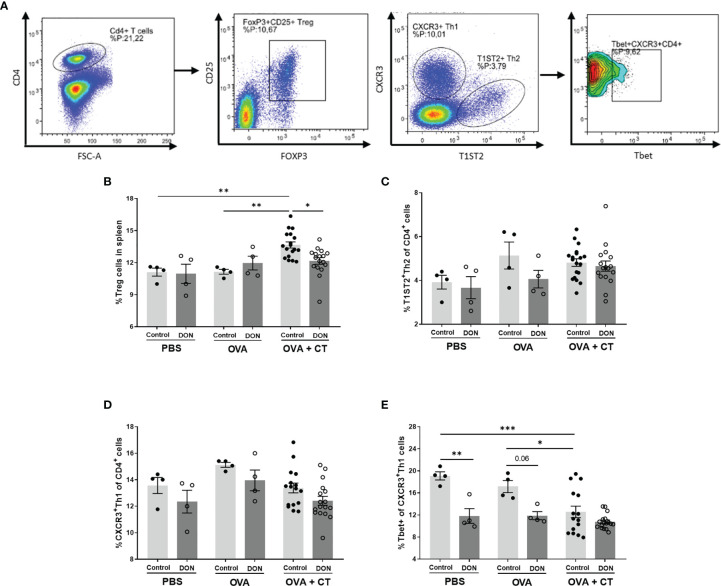
Maternal deoxynivalenol (DON) exposure modulated the splenic T cell populations in the female offspring. Pregnant mice fed either a control or DON-contaminated diet (12.5 mg/kg) during pregnancy and lactation period. Female offspring received oral sensitizations with either phosphate-buffered saline (PBS), ovalbumin (OVA), or OVA with cholera toxin (CT) after weaning. A week after the last sensitization, female offspring were sacrificed and spleen samples were used for flow cytometric analysis of T cell subpopulations. **(A)** Gating strategy for selecting different subtypes of T cell out of live CD4^+^ cells in spleen. Percentages of **(B)** CD25^+^Foxp3^+^ regulatory (Treg) cells; **(C)** T1ST2^+^ Th2 cells; **(D)** CXCR3^+^ Th1 cells; and **(E)** Tbet^+^ cells out of CXCR^+^ Th1 cells; Data are presented as mean ± SEM. *p < 0.05, **p < 0.01 and ***p < 0.001 indicate statistical differences. [FOXP3 (fork-head box P3), CXCR3 (C-X-C Motif Chemokine Receptor 3), T-bet (T-box expressed in T cells, TBX21)].

The frequency of regulatory T cells (Treg) and T helper cells (Th1 and Th2) in isolated spleen samples were studied in both vaccination and allergy models, using flow cytometry (**Figure 4A**, gating strategy). In the OVA-induced food allergy model, after oral OVA challenge, OVA+CT sensitization in the offspring of non-exposed mice significantly increased the percentage of Treg cells, compared to PBS and OVA-sensitized groups (**Figure 4B**, p<0.01), whereas no significant increase was detected in OVA+CT-sensitized group from DON-exposed mothers. Splenic Treg population in food allergic animals born to DON-treated mice was significantly lower than allergic mice from control mothers (p<0.01). No significant differences on percentages of T1ST2^+^ Th2 cells and CXCR3^+^ Th1 cells were observed between treatment and dietary groups (**Figures 4C, D**). However, CT+OVA sensitization followed by OVA challenge causes a significant decrease in Tbet^+^ Th1 cell population in mice born to control mothers (**Figure 4E**, p<0.05 and p<0.01 compared to control and OVA groups, respectively). A similar level of reduction in Tbet^+^ Th1 cell population was observed in all treatment groups from the offspring of DON-exposed mothers.

**Figure 5:**
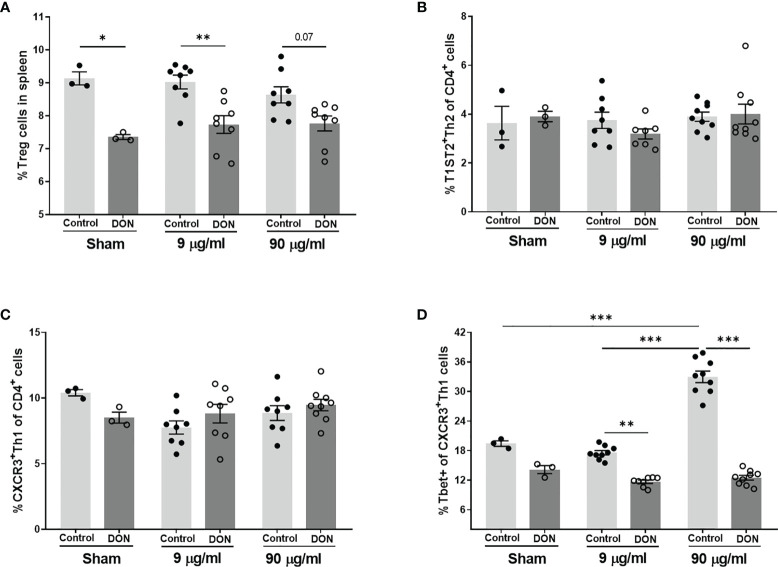
Maternal deoxynivalenol (DON) exposure modulated the splenic T cell populations in the male offspring. Pregnant mice fed either a control or DON-contaminated diet (12.5 mg/kg) during pregnancy and lactation period. Male offspring received subcutaneous injections of either phosphate-buffered saline (PBS, sham) or Influvac (9 and 90 µg/mL) after weaning. 10 days after the second injection, male offspring were sacrificed and spleen samples were used for flow cytometric analysis of T cell subpopulations. Percentages of **(A)** CD25^+^Foxp3^+^ regulatory (Treg) cells, **(B)** T1ST2^+^ Th2 cells; **(C)** CXCR3^+^ Th1 cells, and **(D)** Tbet^+^ cells out of CXCR^+^ Th1 cells, out of live CD4^+^ cells in spleen; Data are presented as mean ± SEM. *p < 0.05, **p < 0.01 and ***p < 0.001 indicate statistical differences. [FOXP3 (fork-head box P3), CXCR3 (C-X-C Motif Chemokine Receptor 3), T-bet (T-box expressed in T cells, TBX21)].

Similar results were observed in the splenic T cell populations of vaccinated animals (**Figure 5**). Vaccination with different concentrations of Influvac had no significant effect on Treg cells, however significant reduction of splenic Treg cells was observed in all treatment groups of the offspring of DON-treated mice (**Figure 5A**). Although no significant differences for Th2 and Th1 cell populations were observed between treatment and dietary groups (**Figures 5B, C**), vaccination with higher concentration of Influvac significantly increased Tbet^+^ Th1 cells in the spleen of the offspring born to control mice (**Figure 5D**). However, in animals born to DON-exposed mothers no significant increase in Tbet^+^ Th1 cell after vaccination was detected.

### 3.5 Maternal DON-Exposure Decreases *Ex Vivo* Th1-Related Cytokine Production

**Figure 6:**
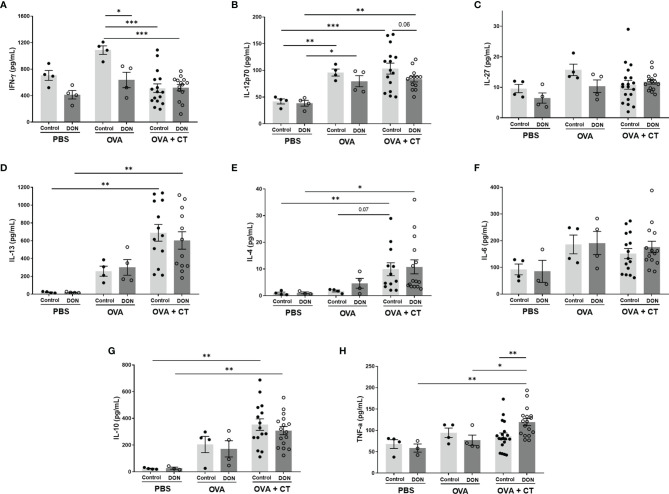
Maternal deoxynivalenol (DON) exposure modulated the cytokine production of ovalbumin (OVA)-stimulated splenocytes of female offspring *ex vivo*. Pregnant mice fed either a control or DON-contaminated diet (12.5 mg/kg) during pregnancy and lactation period. Female offspring received oral sensitizations with either phosphate-buffered saline (PBS), OVA, or OVA with cholera toxin (CT) after weaning. A week after the last sensitization, female offspring were sacrificed and collected splenocytes were re-stimulated *ex vivo* with OVA. **(A–H)** Interféron (IFN)-γ, interleukine (IL)-12p70, IL-27, IL-13, IL-4, IL-6, IL-10 and tumor necrosis factor (TNF)-α concentrations. Data are presented as mean ± SEM. *p < 0.05, **p < 0.01 and ***p < 0.001 indicate statistical differences.

The antigen-specific cytokine expression from splenocytes isolated from the offspring were evaluated by *ex vivo* re-stimulation of the cells with either OVA in allergy model, or Influvac-treated DCs in vaccination model. OVA-specific IL-6 and IL-27 release were not affected by maternal DON-exposure or sensitization status of the offspring (**Figures 6C, F**). Although sensitization with OVA+CT followed by OVA challenge induced significant elevation in IL-12p70, IL-13, IL-4 and IL-10 release, the release of these cytokines was not further affected by maternal exposure to DON (**Figures 6B, D, E, G**). In the offspring of control mothers, IFN-γ release in splenocyte supernatant was significantly lower in the offspring receiving OVA+CT, compared to OVA-sensitized group (p<0.001), whereas in the offspring of DON-exposed mothers the concentration of IFN-γ was as low as OVA+CT sensitized mice (**Figure 6A**). Maternal exposure to DON also significantly increased TNF-α release from cells of OVA+CT sensitized mice born to DON-exposed mothers, compared to the cells of animals from control-fed mothers (**Figure 6H**).

**Figure 7:**
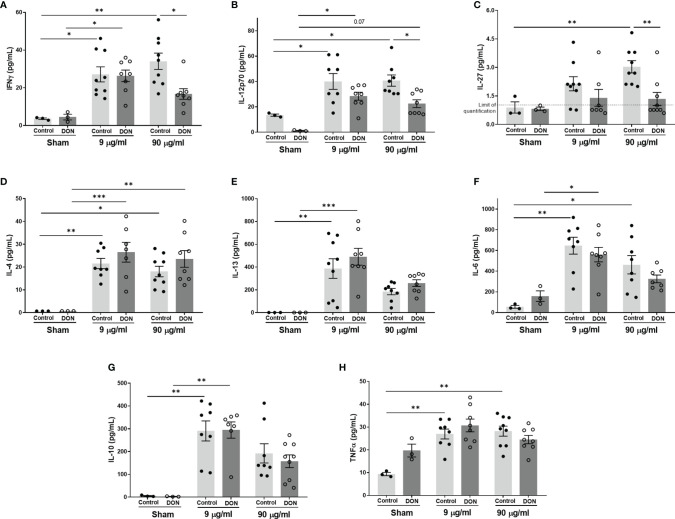
Maternal deoxynivalenol (DON) exposure modulated the cytokine production of splenocytes of male offspring co-cultured with influenza-loaded bone marrow-derived murine dendritic cells (DCs) *ex vivo*. Pregnant mice fed either a control or DON-contaminated diet (12.5 mg/kg) during pregnancy and lactation period. Male offspring received subcutaneous injections of either phosphate-buffered saline (PBS, sham) or Influvac (9 and 90 µg/mL) after weaning. 10 days after the second injection, male offspring were sacrificed and collected splenocytes were co-cultured with influenza-loaded bone marrow-derived DCs from healthy, untreated mice. **(A–H)** Interferon (IFN)-γ, interleukine (IL)-12p70, IL-27, IL-13, IL-4, IL-6, IL-10 and tumor necrosis factor (TNF)-α concentrations. Data are presented as mean ± SEM. *p < 0.05, **p < 0.01 and ***p < 0.001 indicate statistical differences.

In male offspring born to control-fed mice, production of all tested cytokines from isolated splenocytes were significantly increased in vaccinated groups (**Figure 7**). The effect of the higher dose of Influvac was more prominent in release of IFN-γ and IL-27, while the effect of the lower dose was more prominent on IL-13, Il-6 and IL-10. Maternal DON exposure had no significant effect on release of IL-4, Il-13, IL-6, IL-10 and TNF-α by splenocytes from vaccinated mice, compared to the mice born to control mothers, however when comparing groups vaccinated with the higher dose of Influvac, splenocytes of mice from DON-fed mothers had a significantly lower production of Th1-related IFN-γ, IL-12p70 and IL-27 (**Figures 7A–C**).

## 4 Discussion

There are increasing evidence supporting the significant role of immunotoxicity in development of non-communicable diseases (6). Therefore, studying early life toxicity induced by regularly occurring immunotoxins, such as mycotoxins, is of greatest importance. DON, a highly prevalent food contaminant, is a potent immunotoxic agent but information on its effects on immune development in early life is scarce. Administration of 4.42 mg DON/kg diet to pregnant pigs during second half of pregnancy was shown to induce no significant histomorphological changes in the lymphoid organs of the fetuses (40). However, intravenous administration of DON (300 µg in 500 mL of infusion) in pregnant pigs at the end of gestation altered the prevalence of different lymphocyte subtypes in the blood of piglets after birth (27), indicating a disturbed and unbalanced immune system in these animals. The present study is the first to investigate the consequences of developmental immunotoxicity of DON in later stages of life.

The allergic immune response to OVA was studied in female offspring born to either control or DON exposed mothers. When investigating OVA+CT-sensitized animals, significant increases in ASR and average anaphylactic shock scores upon intradermal challenge were observed in the offspring born to DON-exposed mothers as compared to control mothers, indicating that these animals had more severe allergic reactions. OVA-specific serum IgGs and IgE levels were more elevated in allergic mice born to DON-fed animals, which further confirms the higher intensity of OVA-specific allergic immune responses due to maternal exposure to DON. Murine MCP-1 is another serum marker for mast cell-dependent intestinal inflammation in murine allergy models, and can alter the permeability of the small intestine by breaking intercellular tight junction connections (41). Production of mMCP-1 was increased in OVA+CT-sensitized mice, however maternal DON-exposure had no significant effect on soluble mMCP-1 levels. Interestingly, when comparing OVA-treated animals (without CT), an increase in ASR and serum IgGs production after OVA challenge was detected in mice born to DON-exposed mothers, though the level of OVA-specific IgE levels were not affected. This observation indicates that these animals might be more prone to develop allergy. Treatment with the mucosal adjuvant CT disrupts the intestinal epithelial cell layer and facilitates allergy development to OVA (24), while OVA treatment without CT is not expected to induce significant allergic reactions. Therefore, these observations indicate that early-life DON exposure can facilitate allergy development in the absence of CT. Larger sample size for OVA-treated group is required for definite conclusion, however our results indicate that early life exposure to DON worsens the OVA-sensitization response in mice.

Results of an earlier study on adult mice showed that DON can mimic the effect of CT in allergic sensitization (24). Oral administration of DON together with whey protein facilitated food allergy induction, indicated by increased ASR values and serum levels of whey-specific IgE, and the production of IL-5 and IL-13 in re-stimulated splenocytes (24). A possible mechanism responsible for increased susceptibility to develop allergy after direct exposure to DON could be the induction of epithelial cell stress and intestinal barrier disruption, through changing the expression of junctional proteins, leading to increased intestinal permeability (24, 37). In the current study comparing the mRNA expression of junctional proteins in the duodenum of mice born to DON-exposed mothers to those born to control mothers revealed no significant difference. These observations might be in part due to the indirect exposure of the offspring to DON. Furthermore, the offspring received control diet from a week before weaning till the end of the experiment; therefore, any modifications in the intestine induced by early exposure to DON could possibly be restored during the DON-free period. Nevertheless, further analyses of protein expression of tight junctions can shed more light on potential DON-induced alterations in the intestine of the offspring.

In a mouse model of house dust mite -induced allergic asthma, acute and subacute oral treatment with DON significantly enhanced the inflammatory responses, by increasing the infiltration of CD3^+^CD4^+^ helper T cells and IgE^+^ B cells and enhancing production of IL-4, IL-5 and IL-13 in local lymph nodes (42). In the current study, there was no significant difference in the population of splenic Th2 cells between different groups, and the production of Th2-mediated cytokines, such as IL-13 and IL-4, from re-stimulated splenocytes of OVA-sensitized animals born to control and DON-exposed mothers was not significantly different. However, production of TNF-α, a pleiotropic cytokine, was increased in OVA-sensitized animals born to DON-exposed mothers, compared to OVA-sensitized mice from control mothers. TNF-α is shown to be required for antigen-specific IgE and Th2-type cytokines production in allergic rhinitis in mice (43), and is necessary for regulating production of IL-4 and IL-13 (44). Furthermore, an overall significant reduction in Tbet^+^ Th1 cells and downregulation of Th1-mediated IFN-γ production was observed in the offspring of DON-exposed mothers in the OVA-specific allergy model. Previous studies have reported that upregulation of Tbet expression directly correlates with inhibition of IL-4-induced IgE and IgG class switching in B cells (45). A reduction of Tbet expression is associated with the development of allergy in mice (34), as a reduction in Th1-mediated immune capacity can disturb the balance between Th1- and Th2-mediated immunity and shift the balance toward Th2-mediated responses, subsequently rendering the immune system more susceptible to allergy development. In addition, it has been shown that higher production of IFN-γ in mice receiving OVA leads to the induction of tolerance, while depletion of IFN-γ results in failure to control Th2 cell responses to allergens (46, 47). Thus, although the percentage of Th2 cells and concentration of Th2-mediated cytokines are not significantly affected by DON, it could be suggested that the increased intensity of allergic reactions and production of OVA-specific immunoglobulins is due to a lack of a proper Th1-mediated immune response which would otherwise neutralize the Th2-mediated allergic responses. However, further investigation with a focus on earlier stages of allergy development can provide a better insight to the mechanisms behind these observations. Similarly, a reduction in Tbet^+^ Th1 cells and Th1-mediated cytokines was observed in OVA-treated mice (without CT) born to DON-exposed mothers, which may, in part, explain the increased ASR and vaccine-specific IgG production in these animals. T cell-mediated mechanisms may play a more prominent role in induction of observed allergic reactions in these animals (48, 49), as the production of IgE in these animals was not significantly affected by maternal DON exposure. However, further investigation with larger sample size is required to confirm these findings and identify potential non-IgE-mediated mechanisms involved in induction of the observed hypersensitivity reactions.

A suppressed immune response to influenza virus antigens was observed in vaccinated mice born to DON-exposed mothers, indicated by a reduced DTH response and serum levels of vaccine-specific IgGs. Similar to the observations in the allergy model, a significant reduction in the percentage of Tbet^+^ Th1 cells in the spleen, and the production of Th1-mediated cytokines, such as IFN-γ, IL-12p70 and IL-27 were observed in these animals. IFN-γ plays a key role in anti-microbial immunity and a decrease in the capacity of IFN-γ production can reduce host resistance to pathogens and increase susceptibility to specific bacterial and viral infections (50, 51). Therefore, lower Th1-mediated responses can, in part, explain diminished vaccine antigen-specific immune responses in the offspring of mice fed with DON-contaminated diet. There is an altered immune response to enteric and respiratory reovirus infection upon DON administration in adult mice (25, 26). Reovirus-specific IgA levels in serum of mice receiving one-time oral gavage of 10-25 mg/kg of body weight DON were elevated and IFN-γ production in response to reovirus was suppressed, which resulted in transiently increased severity of the viral infection (25, 26). Moreover, feeding pigs with DON-contaminated diets (3.5 mg/kg of diet) impaired the immune response after vaccination with PRRSV (porcine reproductive and respiratory syndrome virus) live attenuated vaccine, by decreasing antibody response to vaccination and negatively affecting the replication of vaccinal virus (52). Although the dose of DON, route of administration and exposure duration vary in above-mentioned studies, these observations correspond with the suppressed Th1-mediated immune capacity and Th2-skewing properties of DON.

There was a downregulation of CD25^+^Foxp3^+^ regulatory T cells in the spleen in both allergic and vaccinated mice born to DON-exposed mothers, compared to the offspring of control-fed mothers. A similar decrease in regulatory T cells in the blood leukocytes of piglets was reported after intravenous administration of DON during pregnancy in pigs (27). Regulatory T cells have a prominent role in vaccination responsiveness (53, 54) and allergy development (55). Treg populations actively prevent hypersensitive immune responses (56). CD4^+^CD2S^+^Foxp3^+^ Treg cells can suppress Th2 responses to inhaled and food allergens (57), and it is suggested that the function of Treg cells may be impaired in allergic patients (58). Therefore, a reduced Treg cell population might contribute to the imbalanced immune responses observed in the present study.

This study was designed based on previous experiments with the murine models of pregnancy and OVA-specific food allergy using female C3H/HeOuj mice (34). The OVA-specific food allergy model used in this study was previously optimized using female mice to obtain the desired allergic responses (34, 59), while for vaccination responsiveness murine models are developed using both male and female mice (60, 61). Therefore, female offspring were used for OVA-specific food allergy model and male offspring were used for vaccination model, to obtain the desired immune responses. However, regardless of the immune challenging model, similar Th2-skewing effects were observed in both male and female offspring, thus it can be concluded that the effect of maternal exposure to DON on disturbing the immune balance is consistent in both sexes. Nevertheless, it is indeed highly relevant to further investigate both models in both sexes to compare the sensitivity of male and female offspring to DON-induced effects.

Although there are regulations set to limit the level of DON concentration in human food products (1-2 mg/kg of food) (29), masked and modified forms of DON, which are not detected by established standard methods, may lead to underreporting of mycotoxin levels in food products (62). Moreover, humans are likely exposed to varying levels of the mixtures of mycotoxins present in the different food products on a daily basis, which can have additive to synergistic toxic effects (63, 64), and make it difficult to estimate the exact exposure levels. Thus, investigating the immunomodulatory effect of DON at a wide range of concentrations, including the concentrations above the established limits can be considered as highly relevant. This study focused on the early-life exposure to DON through maternal diet during the entire period of pregnancy and lactation to mimic the real-life human situation, as the exposure to DON through diet is expected to remain similar throughout this period. Nevertheless, studying the effect of DON exposure at specific developmental stages in early life would provide a comprehensive understanding of its mode of action and would help to come up with more effective strategies to prevent DON-induced toxicities.

In conclusion, results of the current study show that early life dietary exposure to the food contaminant DON can adversely influence immune maturation and development during early life in the offspring. As a consequence, the immune system in the offspring is skewed toward an imbalanced state, resulting in an increased allergic immune responses to food allergens and a decreased immune responses to influenza virus vaccine. The observed effects can be, in part, due to the suppressed Th1-mediated immune capacity and Th1/Th2 imbalance toward Th2 in these animals. However, further investigation is required to fully understand the exact mechanisms involved in DON-induced developmental immunotoxicity. Considering the difficulty of eliminating mycotoxin exposure in humans, as well as the impact of climate change on increasing the contamination of agricultural products with fusarium mycotoxins (65), it is important to be aware of the potential harmful effects of DON present in food products and understand the exact mechanisms of its toxicity, in order to efficiently prevent or attenuate its adverse effects.

## Data Availability Statement

The original contributions presented in the study are included in the article/**Supplementary Material**. Further inquiries can be directed to the corresponding author.

## Ethics Statement

The animal study was reviewed and approved by Instantie voor dierenwelzijn, Utrecht University, Utrecht, Netherlands (AVD108002016697).

## Author Contributions

The author’s responsibilities were as follow: BL, SB, AH, and NS: designed the research. BL, SB, AH, JG, AK, and GF: supervised data interpretation. NS and ST: conducted the *in vivo* experiment. NS: analyzed data and wrote the paper. All authors provided critical intellectual input for data interpretation, read and approved the final manuscript.

## Conflict of Interest

The authors declare that the research was conducted in the absence of any commercial or financial relationships that could be construed as a potential conflict of interest.

## Publisher’s Note

All claims expressed in this article are solely those of the authors and do not necessarily represent those of their affiliated organizations, or those of the publisher, the editors and the reviewers. Any product that may be evaluated in this article, or claim that may be made by its manufacturer, is not guaranteed or endorsed by the publisher.
