# Supplementary material for: Exposure to Deoxynivalenol During Pregnancy and Lactation Enhances Food Allergy and Reduces Vaccine Responsiveness in the Offspring in a Mouse Model
Source: Front Immunol. 2021 Dec 17;12:797152. doi: 10.3389/fimmu.2021.797152 (PMC8718709; doi:10.3389/fimmu.2021.797152)
Supplement: Supplementary file 1 [file DataSheet_1.docx]

**Supplementary table 1** List of antibodies used in flowcytometry analysis.

| **Marker** | **Label** | **Catalog number** | **Antibody ID** | **Dilution** |
| --- | --- | --- | --- | --- |
| CD4 | BV510 | 100553 | AB_2561388 | 1:160 |
| CD69 | PE-Cy7 | 25-0691 | AB_469637 | 1:640 |
| T1/ST2 | FITC | 101001F | AB_947549 | 1:200 |
| CXCR3 | PE | 12-1831 | AB_1210734 | 1:100 |
| Tbet | Alexa Fluor647 | 644803 | AB_1595573 | 1:1600 |
| Gata3 | PerCP-eFluor710 | 46-9966 | AB_10804487 | 1:1000 |
| CD25 | PerCP-Cy5.5 | 45-0251 | AB_914324 | 1:1200 |
| CD196 (CCR6) | PE | 129804 | AB_1279137 | 1:640 |
| FoxP3 | FITC | 11-5773 | AB_465243 | 1:100 |
| RorgT | Alexa Fluor647 | 562682 | AB_2687546 | 1:400 |

**Supplementary Table 2**. Number and percentage of animals developing shock symptoms 45 minutes after intradermal skin challenge with ovalbumin.

| **Maternal diet** | **Control** | | | **DON** | | |
| --- | --- | --- | --- | --- | --- | --- |
| Sensitization | **PBS** | **OVA** | **OVA+CT** | **PBS** | **OVA** | **OVA+CT** |
| Mice with  shock score ≥ **1 (%)** | 0/4 (0%) | 0/4 (0%) | 3/15 (20%) | 0/4 (0%) | 2/4 (50%) | 9/15 (60%) |
| Mice with  shock score ≥ **2 (%)** | 0/4 (0%) | 0/4 (0%) | 1/15 (6.6%) | 0/4 (0%) | 0/4 (0%) | 5/15 (33.3%) |
| Mice with  shock score ≥ **3 (%)** | 0/4 (0%) | 0/4 (0%) | 0/15 (0%) | 0/4 (0%) | 0/4 (0%) | 3/15 (20%) |

**Supplementary figure 1.** Body temperature (°C) of female offspring. Pregnant mice fed either a control or deoxynivalenol (DON)-contaminated diet (12.5 mg/kg) during pregnancy and lactation period. Female offspring received oral sensitizations with either phosphate-buffered saline (PBS), OVA, or OVA with cholera toxin (CT) after weaning. A week after the last sensitization, drop in body temperature was determined 45 minutes after intradermal challenge with OVA in the ear. The average body temperature at time 0 before intradermal challenge was 37.7 °C (±0.6 °C), which is determine the graph with dotted line. Data are presented as mean±SEM.

**Supplementary figure 2.** Effect of maternal DON exposure on mRNA expression of intestinal barrier integrity molecules in the offspring. Relative mRNA expression (fold of control, normalized to β-actin) of **A)** zonula occludens-1 (ZO-1), **B)** claudin-4 (CLDN-4), **C)** occludin (OCLD), and **D)** E-cadherin (E-cad). Data are presented as mean±SEM.
